# Fine-mapping and validation of the genomic region underpinning pear red skin colour

**DOI:** 10.1038/s41438-018-0112-4

**Published:** 2019-01-14

**Authors:** Satish Kumar, Chris Kirk, Cecilia Hong Deng, Claudia Wiedow, Mengfan Qin, Richard Espley, Jun Wu, Lester Brewer

**Affiliations:** 1 The New Zealand Institute for Plant and Food Research Limited, Hawkes Bay Research Centre, Havelock North, New Zealand; 2 The New Zealand Institute for Plant and Food Research Limited, Palmerston North Research Centre, Palmerston North, New Zealand; 3 The New Zealand Institute for Plant and Food Research Limited, Mount Albert Research Centre, Auckland, New Zealand; 4 Centre of Pear Engineering Technology Research, Nanjing Agricultural University, 210095 Nanjing, China; 5 The New Zealand Institute for Plant and Food Research Limited, Motueka Research Centre, Motueka, New Zealand

**Keywords:** Genetics, Genomics

## Abstract

Red skin colour is an important target trait in various pear breeding programmes. In this study, the genetic control of red skin colour was investigated in an interspecific population derived using the descendants of the red sport European pear cultivar ‘Max Red Bartlett’ (MRB) and the red-blushed Chinese pear cultivar ‘Huobali’. Approximately 550 seedlings from nine families were phenotyped for red skin over-colour coverage (Ocolcov) and the intensity of red over-colour (Ocolint) on a 0–9 scale, and genotyped using genotyping-by-sequencing. Genome-wide association analyses were conducted using 7500 high-quality single nucleotide polymorphisms (SNPs). Genomic regions on linkage groups (LG) 4 and 5 were found to be associated, and the best SNP (S578_25116) on LG4 accounted for ~15% of phenotypic variation in Ocolcov and Ocolint. The association of S578_25116 with Ocolcov and Ocolint was successfully validated in a sample of ~200 European and Asian pear accessions. The association with red skin at locus S578_25116 was not present in Asian pear accessions, suggesting its close proximity to the MRB’s Cardinal gene. Several putative candidate genes, including MYB transcription factors (*PCP027962* and *PCP027967*), were identified in the quantitative trait locus region on LG4 and await functional validation.

## Crop genetics: What makes a pear red?

Researchers in New Zealand have produced a map of genetic variants linked with red skin color in pears, opening the door to identifying the genes responsible. Satish Kumar and others at the New Zealand Institute for Plant and Food Research Limited measured the skin color of 550 hybrid pear seedlings and sequenced their genomes. Combining these data produced a map of 7,500 variants throughout the genome and identified those associated with red skin color. The most significant variant accounted for about 15% of the color variation. Further analysis of that genomic region revealed several genes which might be related to red skin color. The genomic map produced by this study will improve breeding efficiency by making it possible to screen seedlings for fruit color, but further research is necessary to characterize the candidate genes.

## Introduction

Pear (*Pyrus* sp.) has been cultivated for at least 2000–3000 years, and is currently grown commercially in more than 50 countries in Europe, Northern Africa, Asia, Australasia and North America^1^. Red skin colour, which enhances the appearance of pears and can make them more attractive to potential customers, is considered an important trait to target in breeding programmes around the world, including South Africa, New Zealand, Italy, Spain, China and Australia. Interspecific hybrids are sometimes used in pear breeding programmes to produce new cultivars with novel combinations of colour and texture^2^. Red skin expression in pear results mainly from the concentration and composition of anthocyanin; environmental factors including temperature and UV light can affect anthocyanin concentrations and perceived intensity of colour^3^.

The underlying genetics of anthocyanin biosynthesis and the metabolic pathway have been investigated in various studies and this topic has been extensively reviewed recently^4–6^. Regulation of the anthocyanin pathway in pear is most likely primarily controlled by the MYB-bHLH-WD40 (MBW) transcription factor complex, as it has been demonstrated to be conserved across a broad range of species^7^. For example, in apple, MYB10 and its allelic counterparts have been shown to control anthocyanin expression^4^. In pear, two MYB transcription factors have been associated with anthocyanin production, MYB10 and MYB114, and these appear to act synergistically to enhance pigment concentrations^8^. However, as has been demonstrated across a range of species, anthocyanin biosynthesis may not be regulated by the MBW complex alone^9^. Other transcription factors, such as MADS-box and ethylene response factors (ERF), have also been implicated in pear anthocyanin production^8,10^.

Genetic mechanism behind red skin or blush colour of various European and Asian pear cultivars have been investigated in segregating families. The red skin colour derived from ‘Max Red Bartlett’ (MRB) appears to be controlled by a single dominant gene, whereas the blush colour for ‘Huobali’ may be controlled by complimentary actions of at least two dominant genes^11^. Dondini et al.^12^ reported that the red-skinned phenotype of MRB was controlled by a single dominant gene located on linkage group (LG) 4. Genome-wide dense genotyping, facilitated by the publication of draft genome of Asian^13^ and European^14^ pears, has contributed to the fine mapping of quantitative trait loci (QTL) for colour and fruit quality traits^15^. Using bulked-segregant analysis next-generation sequencing of pooled DNA from red- and green-skinned pear seedlings derived from a cross between red-skinned Asian pear cultivars, Xue et al.^16^ mapped the red/green (R/G) locus to the bottom of LG5.

Previous studies that mapped QTL for pear red skin used a single bi-parental mapping population of small size (~100), and hence lacked statistical power. To address this deficiency, association mapping in multiple family populations, where large numbers of progeny derived from controlled crosses in various mating schemes, has been used for genome-wide association (GWA) studies^17–19^. In this study, we applied GWA analysis in combination with genotyping-by-sequencing (GBS) on a population of genetically related interspecific hybrid families to fine-map the genetic control of red skin colour. Genotype-phenotype associations identified from this GWA approach were validated in an independent population. The identified candidate genes within the QTL region lay a solid base for further functional genomics studies. Furthermore, it is expected that the single nucleotide polymorphic (SNP) markers identified in this study could improve efficiency of pear breeding programmes via marker-assisted selection.

## Material and methods

### Plant material and phenotypes

First-generation families were created at the New Zealand Institute for Plant and Food Research Limited (PFR) in 1986 and 1989 using commercial cultivars of European, Chinese and Japanese pear as parents. MRB, ‘Huobali’ and ‘Worden Seckel’ were the primary sources of red skin/blush. Second-generation families were created in 1996 using the best selections (high proportion of red skin and superior eating quality) produced from first-generation families as parents. Nine third-generation families were created in 2007–2008 using 12 second-generation selections as parents. All 12 parents had ‘PremP45’ (Maxie), a selection from a first-generation family (‘Nijisseiki’ × MRB), in their pedigree. In addition to varying degrees of genetic relatedness among the 12 parents, some of these parents were represented in more than one family (Table 1), which resulted in partial sharing of genomes among hybrid progenies.

**Table 1:** Parentage (each of the 12 colours represents a parent) of the nine interspecific hybrid families, and the parental phenotypes (0–9 scale) for over-colour coverage (Ocolcov) and over-colour intensity (Ocolint)

The segregation ratio of genotypes at the best SNP locus S578_25116 (C/T), and the observed frequency of allele ‘T’ are also shown.

^a^TT genotype was not observed in this family despite both parents a being heterozygous

A random selection of seedlings that reached a minimum height after growing in a field nursery for 1 year, were propagated onto Quince C rootstock, inter-stocked with ‘Beurré Hardy’ and planted in PFR’s orchard in Motueka during 2011. Six fruit harvested from each seedling over two consecutive seasons (2015 and 2016) were stored for 4 weeks at 3 °C, then a further 1 day at 20 °C before assessment. Red skin over-colour coverage (Ocolcov) was scored on a scale from 0 (no red colour) to 9 (completely red). The intensity of red over-colour (Ocolint) was scored on a scale from 0 (none) to 9 (highest).

### DNA extraction and variants discovery from GBS

Protocols for DNA extraction, GBS library preparation and variant discovery were the same as those reported earlier by Kumar et al.^20^. Briefly, GBS libraries were sequenced on the Illumina HiSeq2000 platform and the sequence data were analysed using TASSEL/GBS^21^ pipeline. The fastq files were mapped separately to the *P.* × *bretschneideri* (cultivar ‘Suli’)^13^ and *P. communis* (cultivar ‘Bartlett’)^14^ reference genomes using Bowtie2 (version 2.2.1)^22^ to maximise the number of SNP calls in interspecific hybrid progenies. The various data quality filters used for SNP calling were described earlier by Kumar et al.^20^.

### Linkage map construction

Resulting SNPs from the previous step were used to construct a consensus linkage map. Genotypes of the parents of one family (P449) were not available, hence data from eight families were used for constructing a consensus linkage map. Paternal and maternal maps (16 maps in total) were constructed using *Joinmap* v4.1 software. Then, common markers shared by at least three different paternal (or maternal) maps were selected as a bridge to merge the maps using R package *MergeMap*^23^. Linkage group (LG) identification was determined based on those SNPs common with the previously published map of ‘Old Home’ × ‘Louise Bon of Jersey’^24^.

### Genome-wide association (GWA) analysis

Genome-wide tests for marker-trait associations were conducted using a mixed-linear model approach. A genomic relationship matrix^25^ and principal components (PCs) derived from the genotypic data matrix were used to account for cryptic relatedness and population stratification, respectively. Studies have shown that using genomic (or realised) relationship matrix is more efficient than the pedigree-based relationship matrix to account for Mendelian sampling and segregation distortion^26,27^. Estimate of narrow-sense heritability (*h*^2^) of each trait and genetic correlation between Ocolcov and Ocolint were also obtained. GAPIT^28^ software was used to conduct the above described genetic analyses.

The most significant SNP markers associated with Ocolcov and Ocolint were validated in a germplasm set of about 200 accessions comprising Asian and European pears and their hybrids. More details about this germplasm can be found in Kumar et al.^20^. Searches for potential candidate genes in the vicinity of significant SNPs were also conducted using the Asian^13^ and European pear (double haploid ‘Bartlett’; unpublished) genome assemblies. Candidate gene sequences were then compared to Arabidopsis genome with the Basic Local Alignment Search Tool (BLAST) from The Arabidopsis Information Resource (TAIR**)**^29^ to determine sequence homologs.

## Results

### Genetic analysis of colour

**Fig. 1:**
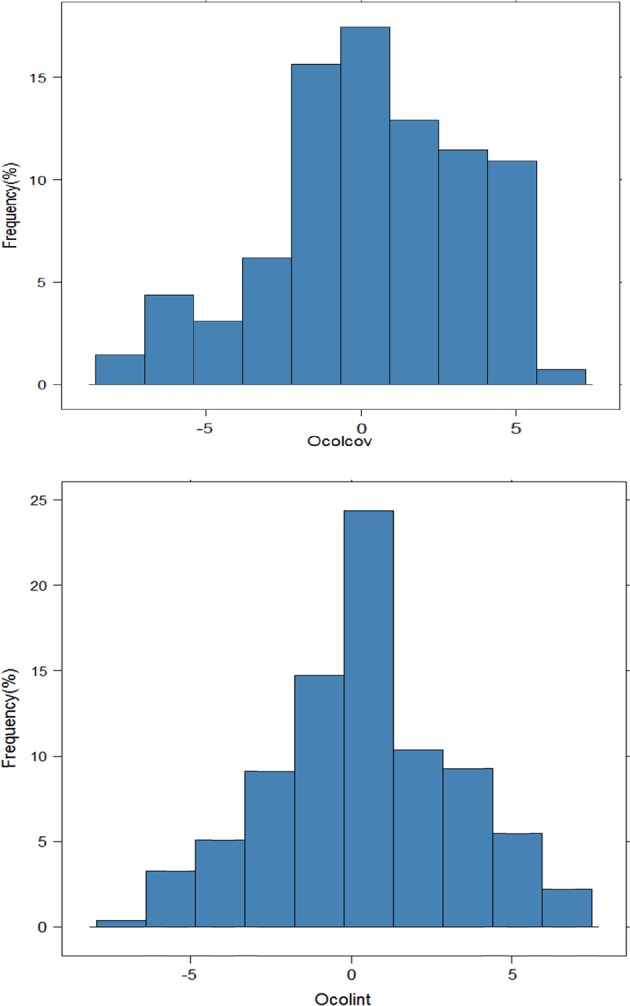
Distribution of over-colour coverage (Ocolcov) and over-colour intensity (Ocolint), after adjusting for fixed effects (e.g. year and assessor), in 550 interspecific pear hybrid seedlings

A total of 550 seedlings with phenotypic and genotypic information were available for genetic analysis (Table 1). The number of seedlings from each family varied between 12 (P484) and 163 (P449). ‘PremP45’, a carrier of a dominant red skin gene of MRB, was represented as a parent or a grandparent in all nine families of this study. Phenotypes of the 12 parents varied considerably; for example, on a 0–9 scale, four parents had Ocolcov and Ocolint scores less than 4, and one parent had a score of >8 for both traits (Table 1). The distribution of trait values, after adjusting for fixed effects, demonstrated the quantitative nature of the red phenotype (Fig. 1). Narrow-sense heritability of Ocolcov and Ocolint was 0.68 and 0.70, respectively, and the estimated genetic correlation between these two traits was about 0.90.

### Genetic map and GWA analysis

Nearly 50,000 SNPs were identified, across nine families, following the earlier described SNP calling criteria. After some additional quality controls (i.e. missing data frequency <10%, minor allele frequency >0.05), over 10,000 SNPs were retained for map construction. A total of about 7500 SNPs could reliably be mapped onto a consensus linkage map spanning about 4100 cM in total. The length of the 17 LGs showed a wide range, from 54 (LG16) to 362 cM (LG13). The average number of markers per LG and the average marker interval were 441 and 0.60 cM, respectively (Supplementary Table S1; Supplementary Figure S1). Overall, there were about 4434 unique genetic positions on this GBS-based consensus map, which means that on average there were 1.7 markers per unique genetic position.

**Fig. 2:**
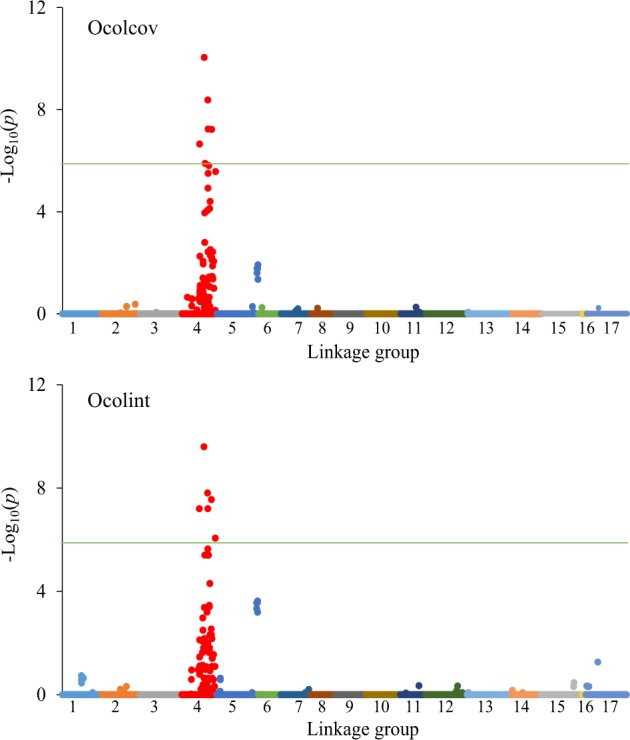
Manhattan plots of the –log_10_(*p*) values for over-colour coverage (Ocolcov) and over-colour intensity (Ocolint) from a genome-wide scan plotted against position on each of 17 *Pyrus* linkage groups (represented by different colours). Green horizontal line indicates the genome-wide significance threshold

The profiles of FDR-adjusted *p*-values (in terms of –log_10_(p)) for all tested SNPs for both traits are illustrated in Fig. 2. A genome-wide *p*-value of <0.01, which roughly equates to a comparison-wise *p*-values of *p* < 1 × 10^−6^ as we tested over 7500 SNPs, was used as a significance threshold for individual SNP testing. The ten most significant SNPs were common to both Ocolcov and Ocolint, and were clustered within a genomic region between 196 and 256 cM on LG4 (Fig. 2). This clustering of significant SNPs suggests the presence of a large-effect QTL on LG4. The most significant SNP (S578_25116), mapped at 196.6 cM on LG4, explained about 15% of phenotypic variation in Ocolcov and Ocolint. For both traits, the Q-Q plots showed a close adherence of the observed and expected –log_10_(*p*) values over most of the range (results not shown). Although not statistically significant, a couple of SNPs (S210_30053 and S210_30038) mapped at the bottom of LG5 and accounted for about 5% of phenotypic variation in Ocolint and Ocolcov, respectively (Fig. 2).

**Fig. 3:**
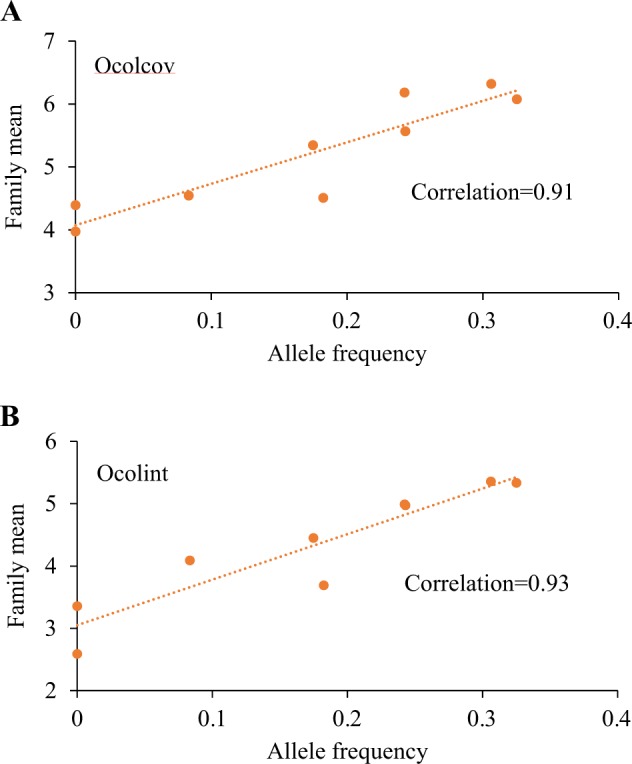
Relationship between frequency of allele ‘T’ at SNP locus S578_25116 (C/T) in a family and family mean for over-colour coverage (Ocolcov) and over-colour intensity (Ocolint) in interspecific pear hybrids

Unexpectedly, the ‘TT’ genotype was not observed in families where both parents were heterozygous (CT) at SNP locus S578_25116 (Table 1). Observed frequencies of the allele ‘T’ at SNP locus S578_25116 varied considerably between families (Table 1), and a high correlation (~0.92) was observed between family performance for Ocolcov and Ocolint and allele frequency (Fig. 3). The average Ocolcov of families P507 and P508 with zero frequency of allele ‘T’ was 4.2 compared with 6.2 for families P493 and P491 with the highest frequency (0.32). Similarly, the average Ocolint varied between 3.0 and 5.3 for families with the lowest and the highest allele frequencies, respectively (Fig. 3).

Separate analyses of various subsets of families identified S39_462595 and S161_15221, which are in close proximity of the overall best SNP S578_25116, as the most significant markers. Joint analysis of families P507 and P508, which did not segregate for S578_25116 (see Table 1), did not show any genomic region significantly associated with skin colour.

### Validation of significant SNPs

**Fig. 4:**
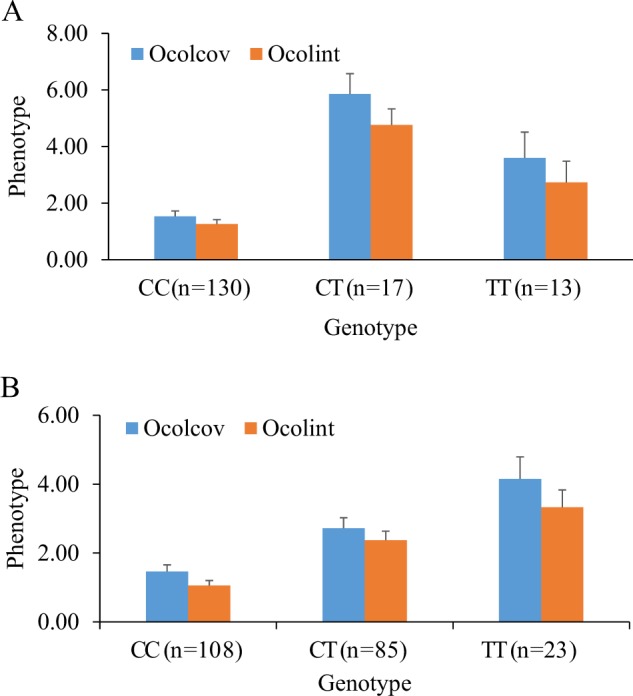
Validation of the effect of best SNPs S578_25116 (**a**) and S123069_330 (**b**) on over-colour coverage (Ocolcov) and over-colour intensity (Ocolint) in an independent population of European and Asian pear accessions. Error bar indicates standard error of mean phenotype (y-axis)

Genotypes from 160 validation samples were available for the most significant SNP (S578_25116), and the estimated frequency of the positive allele in these samples was about 0.10. The number of accessions with genotype CC, CT and TT were 130, 17 and 13, respectively. Accessions with only Asian ancestry did not carry allele T irrespective of their Ocolcov and Ocolint scores. Although, the average Ocolcov and Ocolint of accessions carrying allele T (i.e. with genotype CT or TT) was significantly higher compared with CC genotypes, the pattern of additive association between marker and phenotype was possibly diluted by the small number of accessions with CT and TT genotypes (Fig. 4a). The second best SNP (S123069_330) with somewhat larger sample sizes for CT and TT genotypes, clearly displayed the additive association of allele T with phenotypes (Fig. 4b). Genotypes of some pear cultivars at locus S578_25116 are documented in Supplementary Table S2.

### Candidate genes for large-effect QTL

To identify candidate genes around the major QTL on LG4, searches were carried out on the genomic scaffolds of the most recent versions of *P. communis* and *P*. *bretschneideri* genomes. A list of potential candidate genes in the vicinity of some of the most significant SNPs on LG4 and LG5 are listed in Supplementary Table S3. The largest-effect SNP on LG4 was located on scaffold S578: this scaffold contained 16 gene models and from these, two MYB-related transcription factors (gene ID: *PCP027962* and *PCP027967*) and three E3 Ubiquitin genes (gene IDs: *PCP044630*; *Pbr024602.1* and *Pbr024617.1*) were identified as potential candidates (Supplementary Table S3). The MYB transcription factor genes *PCP027962* and *PCP027967* exhibited sequence similarity to the *Arabidopsis* transcription factor genes *At5G47390* (MYB-like transcription domain) and *At5G35550* (TT2 gene, which encodes a R2R3 MYB domain protein), respectively. The potential candidate genes aligned with the three other scaffolds (S39, S161 and S210) of significance included a family of basic helix-loop-helix (bHLH) transcription factors (e.g. *Pbr009756.1*; *Pbr009770.1*; *Pbr008711.1*; *PCP028905*; *PCP031885* and *PCP014862*) (Supplementary Table S3).

## Discussion

Pear cultivar breeding is a lengthy process, largely because of how long it takes for seedlings to flower (juvenility) and the subsequent, but necessary, time for reliable tree/fruit performance (phenotyping), testing and selection. Decreasing that time can be achieved through reducing the time to fruit, breeding for increased precocity, or using molecular tools^2^. Marker-assisted selection would further improve the efficiency of breeding programmes by enabling screening of young seedlings for red skin/blush colour and elimination of those that do not have the desirable alleles.

In our study, a large number of multi-generation hybrid seedlings with varying degrees of genome sharing with the European and Asian pear cultivars MRB and ‘Huobali’, respectively, presented a mosaic of major and minor genes imparting variation in skin colour. Using advanced genotyping and analysis methods, a cluster of SNPs on LG4 displayed significant association with skin colour. The same set of SNPs were associated with Ocolcov and Ocolint, supporting the observed high genetic correlation (about 0.90) between these traits. We independently validated the additive effect of allele T at SNP locus S578_25116 in a large sample (about 200) of European and Asian pear accessions, adding further credibility to the marker-trait association reported in this study. A moderate performance of P507 and P508 families, which did not segregate for allele T at SNP locus S578_25116, highlighted the contribution of minor genes, especially those inherited from ‘Huobali’, but that might also have come from ‘Worden Seckel’ or ‘Sanguinole’.

The maximum observed frequency of allele T was about 32% despite both parents of a family being heterozygous (CT × CT) (Table 1). The absence of TT genotypes suggested a ‘fitness penalty’ for offspring that were homozygous (TT) for the dominant red allele (T). From these results, we hypothesise that the homozygous dominant form of red skin gene could affect survival of seeds or seedlings, leading to non-Mendelian segregation ratios. Dominant genes underpinning skin colour in some animal species such as mouse were reported to segregate in non-Mendelian ratio, because one-quarter of the offspring (homozygous for the dominant allele) from crosses between heterozygous parents died during embryonic development^30^.

Unlike previous studies, interspecific hybrid seedlings in our study inherited genes for red fruit colour from both European (MRB) and Asian cultivars (‘Huobali’). A previous study^12^ using 90 F1 seedlings of cross ‘Abbé Fétel’ × MRB, mapped the red fruit colour as a morphological marker on LG4 of MRB, which is consistent with results from this study. This dominant gene in MRB is also associated with the red foliage, and was named C, the Cardinal red colour gene^31,32^. In ‘Bayuehong’, a red-blushed cultivar derived from the European pear cultivar ‘Clapp’s Favorite’, a major QTL for red phenotype was mapped to LG5, and a gene *PyMYB114* was identified within the QTL region^8,15^. Xue et al.^16^, using a cross between Asian pear cultivars ‘Mantianhong’ and ‘Hongxiangsu’ (both derived from ‘Huobali’), identified a QTL for red phenotype at the bottom of LG5. A genomic region at the bottom of LG5 identified in our study is consistent with these previous studies^8,16^, and suggests that LG5 could potentially harbour QTLs underpinning red blush development in Asian and European pears as well as interspecific hybrids.

Similar to the apple *MdMYB10* gene, the pear *PcMYB10* gene was mapped by Pierantoni et al.^33^ to the bottom of LG9, and the importance of *MYB10* transcription factors in the expression of red skin phenotype has been widely reported in functional genomics studies^4,5,33–35^. However, there was no significant marker-trait association detected on LG9 in our study, which is consistent with other QTL mapping reports^12,15,16,36^. The molecular basis for this disconnect between QTL mapping and the major anthocyanin-controlling MYB transcription factor is as yet unknown.

Two MYB transcription factors (PCP027962 and PCP027967) were aligned with the scaffold S578 where the most significant SNP (S578_25116) resides. In particular, PCP027962 appears to be homologous to the Arabidopsis MYB-like domain transcription factor (At5G47390), that is reported to be a positive regulator of anthocyanin biosynthesis and is itself regulated by the anthocyanin-associated elongated hypocotyl (HY5) gene^37^. The other gene, PCP027967, is similar to Arabidopsis gene (At5G35550) TT2, which encodes an R2R3 MYB domain protein that acts as a key determinant for proanthocyanidin accumulation^38^. In addition to these MYB transcription factors, the bHLH, E3 Ubiquitin and ABC transporter classes were among the candidate genes identified within the QTL mapping region on LG4 and LG5. Functions of some of these candidate genes have been investigated in various studies^4,5,8,10^. For example, Yao et al.^8^ identified a gene PyMYB114 within the QTL region on LG5, and the transient assays demonstrated its role in biosynthesis of anthocyanins in pear fruits. Taken together with our results, we hypothesise that LG5 also harbours genes underpinning red skin colour development on both Asian and European pears. SNP markers identified in this study will help screening of young seedlings for red fruit colour and enhance the efficiency of pear breeding programmes. More research is required to test the function of candidate genes, especially PCP027962 and PCP027967, identified on LG4 in this study.

## Supplementary information

Supplementary Tables S1, S2 and Fig S1

Supplementary Table S3
