# Supplementary material for: Fine-mapping and validation of the genomic region underpinning pear red skin colour
Source: Hortic Res. 2019 Jan 14;6:29. doi: 10.1038/s41438-018-0112-4 (PMC6331550; doi:10.1038/s41438-018-0112-4)

**Supplementary Table S1**. Number of single nucleotide polymorphisms (SNPs), length and average marker density for each linkage group (LG) of a consensus map constructed from eight interspecific pear families.

| LG | #SNPs | Length (cM) | Average  interval (cM) | Maximum interval (cM) |
| --- | --- | --- | --- | --- |
| 1 | 524 | 242.73 | 0.46 | 5.86 |
| 2 | 508 | 261.63 | 0.52 | 12.49 |
| 3 | 559 | 290.35 | 0.52 | 8.33 |
| 4 | 464 | 296.43 | 0.64 | 10.55 |
| 5 | 552 | 298.25 | 0.54 | 11.93 |
| 6 | 302 | 256.26 | 0.85 | 9.13 |
| 7 | 399 | 228.97 | 0.58 | 5.26 |
| 8 | 325 | 239.04 | 0.74 | 9.34 |
| 9 | 412 | 263.64 | 0.64 | 12.96 |
| 10 | 454 | 227.69 | 0.50 | 6.11 |
| 11 | 335 | 187.14 | 0.56 | 17.66 |
| 12 | 556 | 291.01 | 0.52 | 7.71 |
| 13 | 592 | 362.9 | 0.61 | 18.29 |
| 14 | 389 | 119.72 | 0.31 | 10.97 |
| 15 | 551 | 268.02 | 0.49 | 6.87 |
| 16 | 46 | 54.73 | 1.22 | 6.43 |
| 17 | 541 | 263.68 | 0.49 | 11.77 |

**Supplementary Table S2**. Genotypes of some European and Asian pear cultivars at SNP locus S578_25116. Pb = *Pyrus bretschneideri*, Pc = *P. communis*, Pp = *P. pyrifolia*.

| Cultivar | Species | Genotype |  | Cultivar | Species | Genotype |
| --- | --- | --- | --- | --- | --- | --- |
| ‘Angelys’ | Pc | CC |  | ‘Moonglow’ | Pc | CC |
| ‘Aurora’ | Pc | TT |  | ‘Nellie’ | Pc | CC |
| ‘Autumn Bergam’ | Pc | CC |  | ‘New York’ | Pc | CC |
| ‘Beurré Easter’ | Pc | CC |  | ‘Niitaka’ | Pp | CC |
| ‘Butirra Precoce Morretini’ | Pc | CC |  | ‘Old Home’ | Pc | CC |
| ‘California’ | Pc | TT |  | ‘Ottawa 291’ | Pc | CC |
| ‘Cangxili’ | Pb | CC |  | ‘Ovid’ | Pc | TT |
| ‘Nijisseiki Captain Hardy’ | Pp | CC |  | ‘Packhams Triumph’ | Pc | CC |
| ‘Carmen’ | Pc | CT |  | ‘Passa Crassana’ | Pc | CC |
| ‘Cascade’ | Pc | TT |  | ‘Patten’ | Pc | TT |
| ‘Choju’ | Pp | CC |  | ‘PA2W301’ | Pp | CC |
| ‘Chojuro’ | Pp | CC |  | ‘President D Osmond’ | Pc | TT |
| ‘Colette’ | Pc | CC |  | ‘President Heron’ | Pc | CC |
| ‘PremP35’ | Pp x Pc | CC |  | ‘Qiyuesu’ | Pb | CC |
| ‘Dan Bae’ | Pp | CC |  | ‘Red Sensation Bartlett’ | Pc | CT |
| ‘Doitsu’ | Pp | CC |  | ‘Reimer Red’ | Pc | TT |
| ‘Florida Home’ | Pc | CC |  | ‘Rogue Red’ | Pc | CC |
| ‘Gion’ | Pp | CC |  | ‘Ruby’ | Pc | CC |
| ‘Gold Nijisseiki’ | Pp | CC |  | ‘Seigyoki’ | Pp | CC |
| ‘Gorham’ | Pc | TT |  | ‘Shingo’ | Pp | CC |
| ‘Grand Champion’ | Pc | TT |  | ‘Shinko’ | Pp | CC |
| ‘Hakko’ | Pp | CC |  | ‘Shinsui’ | Pp | CC |
| ‘Harrow Delight’ | Pc | CC |  | ‘Xuehuali’ | Pb | CC |
| ‘Heishi’ | Pp | CC |  | ‘Sierra’ | Pc | CC |
| ‘Highland’ | Pc | TT |  | ‘Starking Delicious’ | Pc | CC |
| ‘Chikusui’ | Pp | CC |  | ‘Starkrimson’ | Pc | TT |
| ‘Hokusei’ | Pp | CC |  | ‘Suisei’ | Pp | CC |
| ‘Hougetsu’ | Pp | CC |  | ‘Swiss Bartlett’ | Pc | CT |
| ‘Howell’ | Pc | CC |  | ‘Tama’ | Pp | CC |
| ‘Harovin Sundown HW606’ | Pc | CT |  | ‘Tenn’ | Pc | CC |
| ‘Hwa Hong’ | Pp x Pb | CC |  | ‘Tosca’ | Pc | CC |
| ‘Imamura Aki’ | Pp | CC |  | ‘Tsuli’ | Pb | CC |
| ‘Jumbo Starks’ | Pc | CT |  | ‘Uvedales St Germaine’ | Pc | CC |
| ‘Jupp’ | Pc | CC |  | ‘Waseaka’ | Pp | CC |
| ‘Margeurite Marrilat’ | Pc | CC |  | ‘Xinyali’ | Pb | CC |
| ‘Max Red Bartlett’ | Pc | CT |  | ‘Ya Li’ | Pb | CC |
| ‘PremP45’ | Pp x Pc | CT |  |  |  |  |
| ‘Merton Pride’ | Pc | CC |  |  |  |  |
| ‘Moders’ | Pc | CC |  |  |  |  |

**Supplementary Fig. S1**. Distribution of SNP markers on 17 linkage groups of the integrated consensus genetic map of interspecific pear families. X-axis indicates linkage group number, and Y-axis length in centiMorgans (cM).


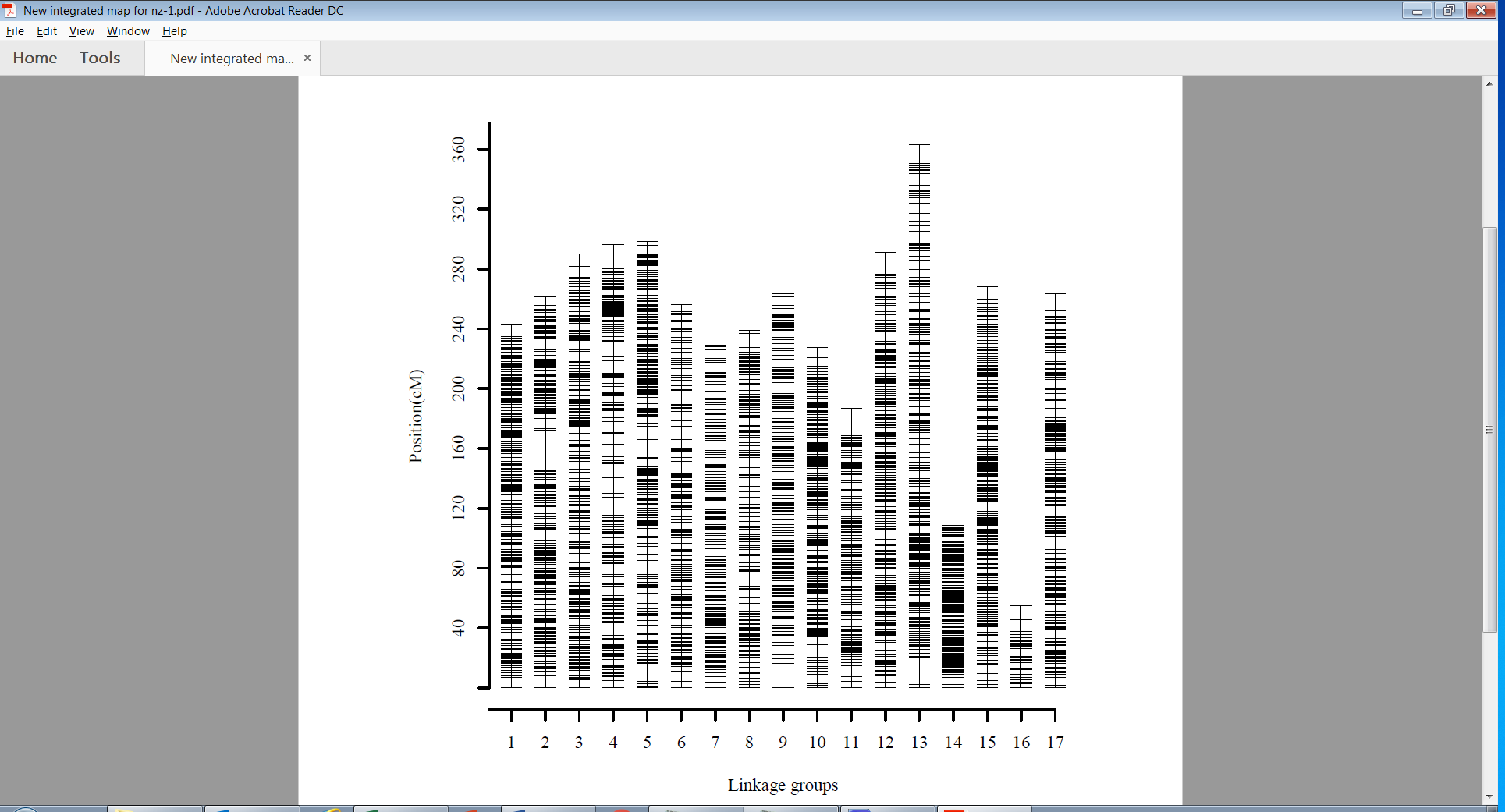

Supplement: Supplementary file 1 — Supplementary Tables S1, S2 and Fig S1 [file 41438_2018_112_MOESM1_ESM.docx]
